# Microbial induced wettability alteration with implications for Underground Hydrogen Storage

**DOI:** 10.1038/s41598-024-58951-6

**Published:** 2024-04-08

**Authors:** Maartje Boon, Ivan Buntic, Kadir Ahmed, Nicole Dopffel, Catherine Peters, Hadi Hajibeygi

**Affiliations:** 1 Delft University of Technology, Faculty of Civil Engineering and Geosciences, 2600 Delft, GA The Netherlands; 2 University of Stuttgart, Institute of Applied Mechanics, Stuttgart, 70569 Germany; 3 University of Stuttgart, Department of Hydromechanics and Modelling of Hydrosystems, 70569 Stuttgart, Germany; 4 NORCE Norwegian Research Centre AS, 5008 Bergen, Norway; 5 Department of Civil and Environmental Engineering, Princeton University, Princeton, New Jersey USA

**Keywords:** Microbiology, Hydrology

## Abstract

Characterization of the microbial activity impacts on transport and storage of hydrogen is a crucial aspect of successful Underground Hydrogen Storage (UHS). Microbes can use hydrogen for their metabolism, which can then lead to formation of biofilms. Biofilms can potentially alter the wettability of the system and, consequently, impact the flow dynamics and trapping mechanisms in the reservoir. In this study, we investigate the impact of microbial activity on wettability of the hydrogen/brine/rock system, using the captive-bubble cell experimental approach. Apparent contact angles are measured for bubbles of pure hydrogen in contact with a solid surface inside a cell filled with living brine which contains sulphate reducing microbes. To investigate the impact of surface roughness, two different solid samples are used: a “rough” Bentheimer Sandstone sample and a “smooth” pure Quartz sample. It is found that, in systems where buoyancy and interfacial forces are the main acting forces, the impact of biofilm formation on the apparent contact angle highly depends on the surface roughness. For the “rough” Bentheimer sandstone, the apparent contact angle was unchanged by biofilm formation, while for the smooth pure Quartz sample the apparent contact angle decreased significantly, making the system more water-wet. This decrease in apparent contact angle is in contrast with an earlier study present in the literature where a significant increase in contact angle due to microbial activity was reported. The wettability of the biofilm is mainly determined by the consistency of the Extracellular Polymeric Substances (EPS) which depends on the growth conditions in the system. Therefore, to determine the impact of microbial activity on the wettability during UHS will require accurate replication of the reservoir conditions including surface roughness, chemical composition of the brine, the microbial community, as well as temperature, pressure and pH-value conditions.

## Introduction

The transition towards renewable energy sources requires large scale energy storage to balance out energy production and demand on seasonal scale, defined by Tera-watt hours (TWh). Large-scale energy can be stored in the form of hydrogen due to its high mass energy density and clean combustion byproducts, but because of its low density, enormous storage volumes are required to store it at TWh scale. Geological porous reservoirs could provide the volume capacity needed for large scale Underground Hydrogen Storage (UHS)^1,2^. As an alternative technology, natural gas (CH_4_) has been stored successfully in subsurface reservoirs for decades. However, important differences between the systems of Underground Gas Storage (UGS) and UHS exist that make the storage of these two gasses not directly translatable. Firstly, the cyclic loading and frequency as well as the purity restrictions of the system will be very different for UHS^3^ than for UGS. Secondly, H_2_ and CH_4_ are very different molecules, as can be seen from the differences in diffusivity, dissolution, and surface/interfacial tension^4^ which can lead to different physiochemical behaviours in the reservoir. Lastly, microbial activity will play a much more important role during UHS, because molecular hydrogen is one of the main electron donors for microbial respiration in the subsurface^2,5–7^.

The subsurface microbial biosphere consists of a high diversity of Bacteria and Archaea from indigenous origin as well as anthropogenically introduced during drilling, pumping and mining. These single-celled organisms play a key role in many of the biochemical and geochemical reactions that take place in geological porous reservoirs which commonly contain high salinity brines with limited nutrients at elevated temperatures and pressures. Despite these harsh conditions, microbial life in these reservoirs is abundant^7,8^ as microbes can exist at a wide range of pressure, salinity, temperature, and pH-value conditions^6^. Next to water of sufficient thermodynamic activity, microbes require an energy source (electron source), an electron acceptor, a carbon source for cell division, and other essential elements and nutrients to be active^6^. They obtain energy by transferring electrons from an electron donor to an electron acceptor. Hydrocarbons and hydrogen are both examples of electron donors that can be used as energy source during microbial respiration in the subsurface.

Microbes can live in the reservoir in planktonic-mode and biofilm-mode. Planktonic microbes are free-living in the reservoir fluid while microbes in biofilm-mode attach to the rock surface and develop a biofilm. Biofilms are communities of aggregated microbial cells embedded in a secreted matrix of extracellular polymeric substances (EPS)^7^. EPS consist of polysaccharides, proteins, nucleic acids, and lipids, with affinity for water varying from hydrophilic to hydrophobic. The overall wettability of the biofilm is mainly determined by the contents of EPS. The biofilm development consists of the following stages: 1. attachment of free microorganisms on a surface, 2. colonization, 3. maturation, and 4. aging and detachment. Whether or not a biofilm forms depends highly on the differences between the wettability of the biomass suspended in the brine and the wettability of the rock surface itself, as this forms the driving force for the attachment^9^. Furthermore, the roughness of the solid surface plays an important role as it can directly control the growth rate, strength and texture of the biofilm. Rough surfaces are favourable for initial adhesion and protection against detachment^9^.

Recently, several studies have discussed microbial activity at UHS sites including analyses of possible hydrogen producing and consuming microbial processes^10^. These studies confirm that the microbial H_2_ utilizing processes of methanogenesis, sulphate reduction, and acetogenesis will likely be most common in UHS sites. Furthermore, an estimation of the risk for microbial growth in 42 depleted oil and gas fields^7^ has been given, as well as reviews of the potential risks for UHS project performance^2,5,6^. All these studies suggest that increased levels of hydrogen, as the results of UHS, could significantly enhance the microbial activity. This could lead to unwanted effects such as hydrogen loss, toxic H_2_S formation, corrosion of the metal infrastructure, and injectivity reduction due to clogging caused by biofilm growth^6,7^. In addition, microbial activity could alter the wettability of the hydrogen/brine/rock system and, consequently, the hydrogen transport behaviour during UHS. This was quantitatively observed in the recent work of Liu et al. 2023^11^ who carried out an experimental pore-scale study, involving a microfluidic porous chip and sulphate reducing bacteria, to investigate microbial impact on hydrogen consumption, contact angle evolution and hydrogen bubble disconnection. Their results showed that microbial activity changed the surface wettability from a water-wet [41^∘^] to a neutral-wet [96^∘^] state compared to a contact angle of [28^∘^] for a sterilized experiment. The observed change in wettability was explained by the adhesion of bioproduct and bacterial cells to the solid surfaces. In addition, it was suggested that the increased pH-value due to microbial growth could also have led to a more hydrophobic system.

Studies investigating the impact of microbial activity on wettability for gas-water systems are scarce. However, numerous studies have investigated the impact of microbial activity on wettability and interfacial tension (IFT) in the subsurface with application for microbially improved oil recovery (MIOR). During the MIOR process, injected bacteria will flow in the water phase, and grow on the oil-water interface where they produce bio-surfactants. These bio-surfactants reduce the oil-water IFT such that the microbes can access the carbon source. This reduction in IFT may in turn change the oil-rock contact angle altering the wettability. In addition, bacteria can attach to the rock surface and eventually form a biofilm, which can lead to further changes in the contact angle. These changes in wettability can significantly enhance oil recovery^12,13^.

Wettability for UHS, in the absence of microbes, has been characterized using several experimental techniques^14^. Static contact angles for the hydrogen/brine/rock system have been measured using the captive-bubble cell approach^4,15^ and in-situ measurements using micro-CT^16^. Advancing and receding contact angles have been measured directly using the tilted plate technique^17,18^ as well as microfluidics^19^, and indirectly by combining capillary pressure measurements with mercury injection capillary pressure (MICP) data^20,21^. These studies showed water-wet^4,15–19,21^ to intermediate-wet^17,18^ conditions with contact angles ranging between 0^∘^ and 76.9^∘^. Water-wet conditions enhance the injectivity of the storage reservoir as hydrogen will preferentially flow through the larger pores, which in turn reduces the trapping potential of the hydrogen gas. Changes in the wettability due to microbial activity from a water-wet to a neutral-wet state, as observed in the literature^11^, could reduce the storage injectivity and increase the amount of hydrogen loss as flow will also occur through the smaller pores and the potential of trapping of the hydrogen gas will increase, both will be unfavourable for UHS.

In this study, the captive-bubble cell approach is used to investigate the impact of microbial activity on wettability for the hydrogen/brine/rock system. Experiments are carried out using “living brine” containing sulphate reducing microbes, and for both a “rough” Bentheimer Sandstone rock slab and a “smooth” pure Quartz slab to be able to investigate the impact of surface roughness on the formation of the biofilm and the observed change in wettability.

## Materials and methods

The impact of microbial activity on the wettability of the hydrogen/brine/rock system is investigated by measuring static contact angles using the captive-bubble cell approach. The experiments are carried out for two different solid substrates: a rough Bentheimer Sandstone slab and a smooth pure Quartz slab. Three different liquids are used: DI water, brine, and living brine. The “living brine” contains the sulphate reducing bacteria “*Oleidesulfovibrio alaskensis*” DSM 17464. The experiments are carried out at the optimum temperature for growth, i.e., 37 ^∘^C, and the pressure of 3 bar. This pressure value is selected based on the maximum pressure (max 5 bar) of the pH-meter which is incorporated into the experimental apparatus to monitor the activity of the “living” brine. To investigate the impact of the presence of metabolites and deadcells in the living brine solution on the wettability of the system, experiments are carried out with the Bentheimer Sandstone for two additional liquids: brine containing just metabolites and no microbes, and a solution of dead cells in DI water. These results are presented and discussed in the Supplementary Information.

### Materials

#### *Bacterial strain*

The sulphate reducing bacteria (SRB) “*Oleidesulfovibrio alaskensis*” DSM 17464 (https://www.dsmz.de/collection/catalogue/ details/culture/DSM-17464) is used as model bacterium in this study. The *O. alaskensis* bacteria strain is a known biofilm former and a relevant strain for subsurface reservoirs as it was isolated from an oil pipeline^22^. It is a gram-negative, non-spore-forming bacteria. It has rod-shaped cells (vibrio) of 1.0–5.0 $\times$ 0.5–1.2 $\mu$m. The bacteria grows at pH-values ranging between 6.5 and 8.5, temperatures between 10 ^∘^ and 45 ^∘^C, and in brines with salinites ranging from 0 to 10% (w/v) NaCl. The optimum conditions for growth are at 37^∘^ C, at pH-value 7.0, and 2.5% (w/v) NaCl. A carbon source is required, which can be acetate. The respiration is strictly anaerobic and reduces sulphate^23^. It preferably reduces lactate but in the absence of lactate other electron donors can be used in the respiration process, including H_2_.

#### *Rock samples*

The experiments are carried out using a Bentheimer Sandstone rock slab and a smooth pure Quartz slab with surface roughness values of 0.03 mm and 0.003 mm, respectively. The Quartz sample was polished with a felt cloth using a paste containing monocrystalline diamonds (DP-Paste M, 3 $\upmu \hbox {m}$). Both samples have dimensions of 30$\times$6$\times$12 mm. The Bentheimer Sandstone rock slab is cut from the same Bentheimer Sandstone block as the samples used in Hashemi et al. 2021b^4^, 2022^15^ and more detailed information about the rock properties can be found there.

#### *Gas and liquids*

The H_2_ gas used in the experiment consists of 99.99 mol % purity H_2_ produced by Linde-gas Company. The density of the gas at the experimental conditions of T=37 ^∘^C and P=3 bar is 0.234 kg/$\text {m}^3$. Three different liquids are used for the contact angle measurements: DI-water, brine, and living brine. The density values of DI-water and brine at the experimental conditions of T=37 ^∘^C and P=3 bar are 993.4 kg/$\text {m}^3$ and 1010.2 kg/$\text {m}^3$, respectively. The brine mineral components are listed in Table 1. Note that the brine contains plenty of sulphate and several nutrients. To create the media for growing the living brine, 48 mM lactate, 20 mM acetate, vitamins and trace elements are added to the brine recipe. Furthermore, a buffer solution (Na_2_CO_3_ 1.5 g/liter media) is added to adjust the pH-values to 7.0 (https://www.dsmz.de/microorganisms/medium/pdf/DSMZ$\_$Medium195c.pdf). Before adding the sulphate reducing bacteria, the brines are sterilized by autoclaving (20 min at 120 ^∘^C). Finally, the media is inoculated with 40 ml of stock culture of the SRB using a syringe. This process is carried out at 37 ^∘^C in a nitrogen filled glovebox to create an anaerobic environment and ensure that the medium stays anoxic. Before being used in the experiments, the living brine undergoes a SRB enrichment process. This process takes approximately 3 days during which the SRB grow at a very fast rate, using up all the lactate while producing H_2_S. During the experiment the living brine will live on sulphate and use H_2_ as electron donor and acetate as a carbon source. The reactions which are expected to take place during the experiment are

1 $$\begin{aligned} SO_{4}^{2-} + 4H_2 + H^+ \rightarrow HS^- + 4H_2O \end{aligned}$$

2 $$\begin{aligned} HS^- + H^+ \leftrightarrow H_2S \end{aligned}$$

3 $$\begin{aligned} HS^- + Fe^{2+} \rightarrow FeS + H^+ \end{aligned}$$

It can be seen that, next to the formation of H_2_S gas, the formation of iron sulfide deposits is to be expected. The Fe(II) for this reaction is present in the media as part of the trace element solution. Moreover, the Bentheimer rock contains some iron-(hydr)oxides^24^ which could also provide Fe(II). To get an indication of the fluid-fluid interfacial tensions of the sytem interfacial tensions were measured at ambient conditions for the DI-water/air (72 mN/m), brine/air (71 mN/m), solution of dead cells in DI-water/air (52 mN/m), and brine with metabolites but without microbes/air (55 mN/m). The interfacial tension of DI-water/hydrogen at ambient conditions is 72.3 mN/m^25^ which is similar to that of DI-water/air. Therefore, similar differences in interfacial tension values are expected when these liquids are in touch with H_2_ instead of air.

**Table 1:** Brine ingredients.

Chemical	Amount	Unit
Na_2_SO_4_	3.00	g
KH_2_PO_4_	0.20	g
NH_4_Cl	0.30	g
NaCl	21.00	g
MgCl_2_ x 6H_2_O	3.00	g
KCl	0.50	g
CaCl_2_ x 2H_2_O	0.15	g
DI water	920.00	ml

### Methods

#### Captive-bubble cell approach

**Figure 1:**
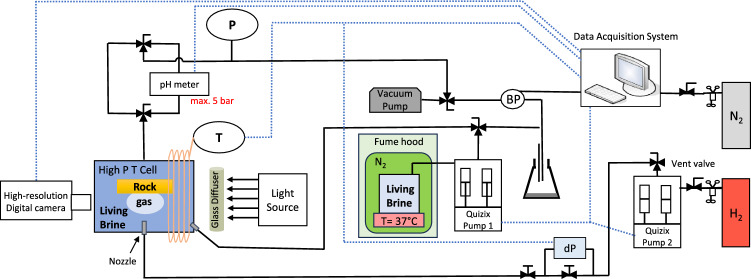
Experimental apparatus used for the experiments.

The captive-bubble cell approach is used to investigate the impact of microbial activity on the wettability of the H_2_/brine/rock system by measuring the contact angle between the rock-brine interface and the brine-hydrogen interface. The experimental apparatus consists of a high pressure/high temperature titanium cell with an inner volume of 10 ml. A solid sample (Bentheimer Sandstone and pure Quartz) is placed in the center of the cell. The bottom of the cell is connected with two continuous flow pumps, one filled with liquid and one filled with hydrogen. To completely fill the cell with DI-water and to ensure that no gas bubbles will stay behind, the outlet located at the top of the cell is initially connected to a vacuum pump while DI-water is injected slowly from the bottom of the cell. After the cell is completely filled with DI-water, the outlet at the top of the cell is connected to a back-pressure regulator to control the pressure in the cell. A heating tape is wrapped around the cell to control the temperature in the cell which is kept at 37^∘^C during the entire experiment as this is the optimum growth temperature for the bacterial strain used in the experiment. A pH-meter (Knauer CM 2.1S with the accuracy of ±0.5 and the precision of ±0.2) is placed between the cell and the back-pressure regulator to be able to continuously measure the pH-values. The pH-value is a direct indicator of the activity of the living brine. The maximum pressure of the pH-meter is 5 bar. For this reason the experiments are carried out at an ambient pressure of 3 bar. A schematic of the experimental apparatus can be seen in Fig. 1. The experimental apparatus is placed next to a fume hood in which a nitrogen filled glove box is placed where the living brine is grown and kept at a temperature of 37^∘^ C. The temperature is regulated by placing the glass living brine container on top of a heating plate. During the growth of the living brine the Fe(II) from the media can react with the H_2_S formed by the microbes, resulting in black iron-sulfide particles. A 10 micrometer filter is placed on the inlet tubing of the liquid pump to ensure that no iron-sulfide particles will enter the pump. For both solid samples (Bentheimer Sandstone and Quartz) contact angles are measured for DI-water and living brine. In addition, for the Bentheimer Sandstone sample contact angles are also measured for brine. For each measurement, a gas bubble of approximately 2 mm in diameter is released from a nozzle at the bottom of the cell into the liquid. The bubble will rise due to buoyancy until it reaches the surface of the solid sample. The bubble slowly dissolves and diffuses into the liquid until it disappears. This takes on average 2 hours depending on the saturation state of the liquid. The dissolution rate of the bubble does not affect the contact angles^15^. Images of the dissolving bubble are taken every minute with a resolution of 3216$\times$2136 pixels using a Nikon D90 camera with a Laowa lens (100mm F2.8). The camera is connected with the software “Camera Control Pro 2”. For each of these images the contact angle and volume are determined as described in the Image Analysis section. In this way, for each measurement, a wide range of bubble volumes are analyzed and the corresponding contact angles are determined. The accuracy range of the contact angles measured using this technique is ±3 ^∘^^15^. The pressure and temperature in the cell are continuously monitored. The pH-value is recorded at the start of each day. An overview of the experimental conditions during each measurement, including pH-value and liquid flow rate, can be found in Table 2.

**Table 2:** Experimental conditions for the Bentheimer Sandstone and Quartz systems.

Time	Liquid	Flow rate (ml/min)	pH	Location wrt nozzle
Bentheimer Sandstone				
	DI			Center
	Brine			Center
Day 2	LB	0.02	6.6	Right
Day 4	LB	0.02	7.1	Center
Day 9	LB	0.02	7.7	Right
Day 15	LB	0.0005	8.2	Right
Day 22	LB	na	na	Center
Day 39	LB	0.0005	8.8	Right
Quartz				
	DI			Center
Day 1	LB	na	6.6	Center (left)
Day 7	LB	0.05	7.3	Center
Day 10	LB	0.05	7.4	Center (right)
Day 16	LB	0.05	7.2	Right
Day 21	LB	0.05	8.6	Left
Day 31	LB	0.002	8.5	Left

#### Image analysis

Contact angles and volume are derived for each of the images taken during the experiment using the Axisymmetric Drop Shape Analysis-Profile (ADSA-P) technique^26^. The ADSA-P technique is defined for gravity-capillary equilibrium and is based on the Young-Laplace equation. It fits the best theoretical Laplacian curve on the physical observed bubble interface. To calculate the volume of the bubble, the outer diameter of the nozzle is used. For more details about the image analysis procedure the reader is referred to Hashemi et al. 2021^4^ and Hashemi et al. 2022^15^.

#### SEM imaging

The solid surface is imaged before and after each experiment using Scanning Electron Microscopy (SEM). A FEI Quanta 650 FEG scanning electron microscope (SEM) is used for this purpose which is connected to an Energy Dispersive Spectroscopy (EDS) detector. The images are taken in Concentric BackScatter mode. For the first sample (Bentheimer Sandstone), we only aim to identify whether microbial activities were present during the experiment. As such, the SEM imaging technique following the standard procedure was used, as it allows for identification of the microbial activity by detecting some traces of the formation of biofilm. For the second experiment (pure Quartz sample), however, it was also aimed to study the structure of the fairly intact biofilm. Therefore, the following preservation procedure was carried out immediately after taking the sample out of the captive-bubble cell: (1) Place the sample in 2.5% glutaraldehyde in the fridge at 4^∘^ to 7^∘^ C overnight to fix the biofilm on the sample, (2) Wash the sample with water twice, (3) Let the sample air dry overnight, and (4) Sputter the sample with gold. For the gold sputtering a Leica EM SCD 500 sputter coater is used.

## Results and Discussion

**Figure 2:**
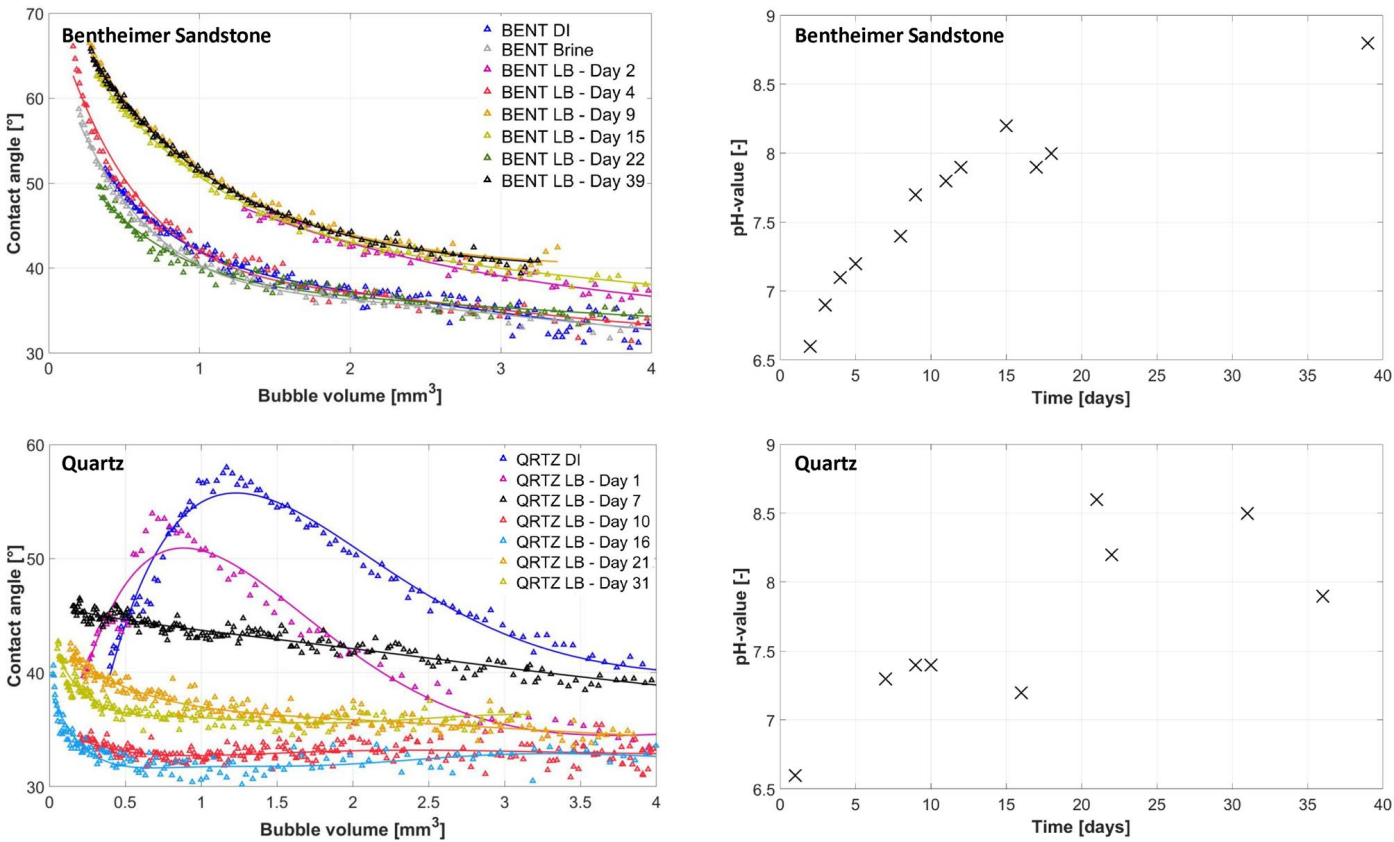
Contact angle versus volume (left) and pH-value vs time (right) for the H_2_/living-brine/Bentheimer-Sandstone system (top) and H_2_/living-brine/Quartz system (bottom).

**Figure 3:**
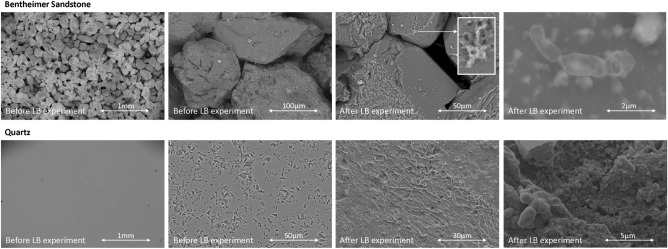
SEM images before and after the Living Brine (LB) experiment for the Bentheimer Sandstone sample (top) and Quartz sample (bottom).

Two wettablity alteration mechanisms due to microbial activities are expected to take place in these experiments: 1) reduction of the interfacial tension due to the presence of microbial (dead) cells and metabolites, and 2) the formation of biofilm consisting of EPS. The effect of the reduction in interfacial tension will be observed as an immediate effect, while the formation, maturation and possible detachment of the biofilm will change the wettability over time. Figure 2 (top) shows the apparent contact angles versus the hydrogen bubble volume and the pH-value versus time for the living brine experiment using the Bentheimer Sandstone rock slab, pure H_2_ gas, and brine enriched with sulphate reducing microbes. The total duration of the experiment is 39 days (the batch of living brine ran out after 39 days). In addition, the apparent contact angles for DI-water and brine measured before the start of the living brine experiment are shown which range between 30 ^∘^  and 60 ^∘^ indicating water-wet conditions. This is in agreement with previously reported contact angles in the literature for the hydrogen/brine/rock system^4,15–19,21^. It can be seen that the contact angles of the living brine experiment on day 2 slightly increase with approximately 5^∘^ and also the pH-value increases to approximately 7.0. On day 3, the contact angles stay unchanged while the pH-value further increases. Surprisingly, on day 4 the contact angles jump back to the curve of DI-water, while the pH-value keeps on increasing. On day 5, the contact angles jump up again to the values of day 2, and stay unchanged for most of the remainder of the experiment time, while the pH-value rises to values slightly below 9.0. However, on day 22 the contact angles follow the curve of DI-water. A close examination of the bubble images reveals that the curves that follow the DI-water trajectory all correspond to bubbles located straightly above the nozzle, while the curves with the slightly higher contact angles all correspond to bubbles located slightly to the right of the nozzle (Table 2). This suggests that the small changes in the measured contact angles are likely due to the location of the bubble and not due to the microbial activities. Sulfate reduction on H_2_ will increase the pH-value^27^. The observed increase in pH-value with time indicates that the microbes are highly active. More evidence of the activity of the living brine is given by the formation of Iron Sulfide (FeS) which turned the sample very dark. A picture showing a Bentheimer Sandstone sample at the end of a brine experiment next to a Bentheimer Sandstone sample at the end of a “living” brine experiment, which turned completely dark due to the formation of Iron Sulfide, can be found in the Supplementary Material. To investigate whether or not a bio-film had been formed, SEM images were taken after the experiment. The wet Bentheimer sandstone sample was placed in the SEM and dried under vacuum, without following the biofilm preservation procedure as described in the Methods section. This standard SEM procedure without chemical fixation led to disruption of the potential biofilm as we realized. However, traces of biofilm and microbes were still found (Figure 3). For the subsequent sample (Quartz) a more thorough preservation method was used and an intact biofilm could be observed.

Figure 2 (bottom) shows the contact angle versus bubble volume and the pH-value versus time for the living brine experiment using the smooth pure Quartz sample, pure H_2_ gas, and brine enriched with sulphate reducing microbes. In addition, the apparent contact angles for DI-water before the start of the living brine experiment are shown which range between 40 ^∘^  and 60 ^∘^ indicating water-wet conditions. This is in agreement with previously reported contact angles in the literature for the hydrogen/brine/rock system^4,15–19,21^. The living brine experiment using the pure Quartz sample lasted for 31 days (the batch of living brine ran out after 31 days) and showed very different behaviour compared to the experiment with the Bentheimer Sandstone sample. For one, the initial shape of the curves are different. While contact angles monotonically increased with decreasing volume for the Bentheimer Sandstone, it was not the case for the pure Quartz sample. Here, the contact angles for DI-water initially increased with decreasing volume up to a volume of around 1-1.5 mm^3^ after which the contact angle started to decrease again. The contact angles measured on day 1 of the living brine experiment using the Quartz sample showed the same behaviour. However, the contact angle values were slightly lower compared to DI-water. This is likely the result of the change in the interfacial tensions ($\sim$70 mN/m for DI-water and $\sim$50 mN/m for living brine), which according to the sensitivity analysis presented in the literature^15^ should lead to a decrease in contact angle of [$\sim$6 ^∘^] at a bubble volume of  4 mm^3^ (r=1 mm). Clearly the impact of the difference in interfacial tension between DI-water and living brine on the apparent contact angle was not observed in our study for the Bentheimer Sandstone sample. This is likely caused by the high surface roughness value of the Bentheimer Sandstone. The study of Xiao et al. 2022^28^, looked at the impact of surface roughness on the apparent contact angles of bubbles for a wide range of intrinisic wettabilities. They observed that for rough surfaces, like the Bentheimer sandstone in this study, similar apparent contact angle are observed for a wide range of intrinsic contact angles, while for smooth surfaces the apparent contact angles do change significantly when the intrinsic wettability changes (also see Supplementary Information).

During the living brine experiment with the Quartz sample, the contact angle values at a bubble volume of 1mm^3^ decreased from $\sim$50^∘^  at day 1 to $\sim$35^∘^  at day 31, and also the shape of the contact angle versus volume curve changed and became more similar to the curves obtained for the Bentheimer Sandstone experiment, albeit with lower contact angle values. The pH-value increased during the experiment starting from 6.6 at day 1, to values around 8-8.5 at the end of the experiment. This increase in pH-value indicates high microbial activity and the observed changes in contact angle suggests that a biofilm has formed on the Quartz surface. To visualize the biofilm, first the biofilm preservation procedure was carried out as described in the Methods section before placing the sample in the SEM for imaging. A full grown biofilm was observed as can be seen from the images in Fig. 3.

**Figure 4:**
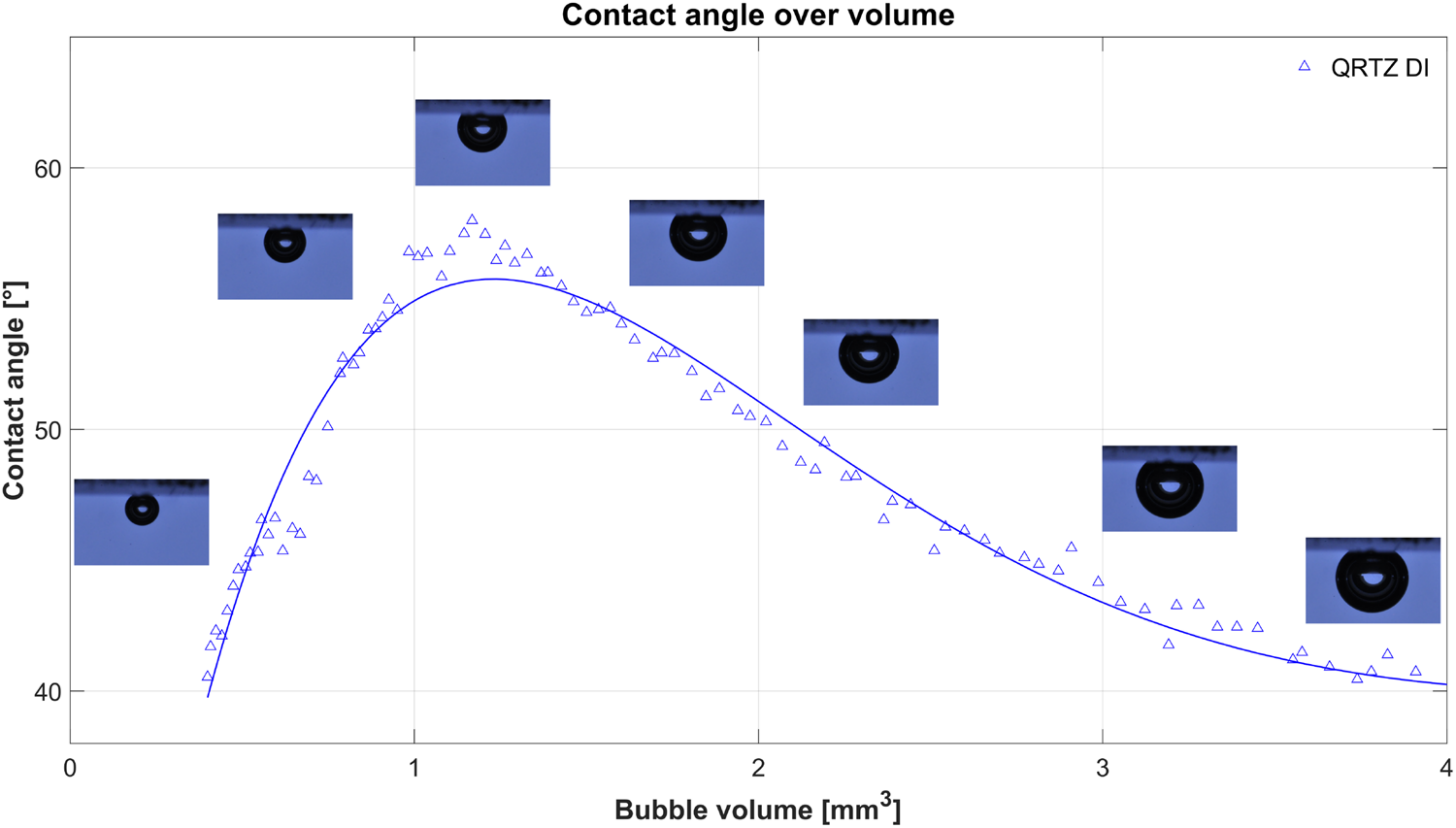
Contact angle versus volume graph for hydrogen bubbles in touch with a pure Quartz sample and DI-water.

**Figure 5:**
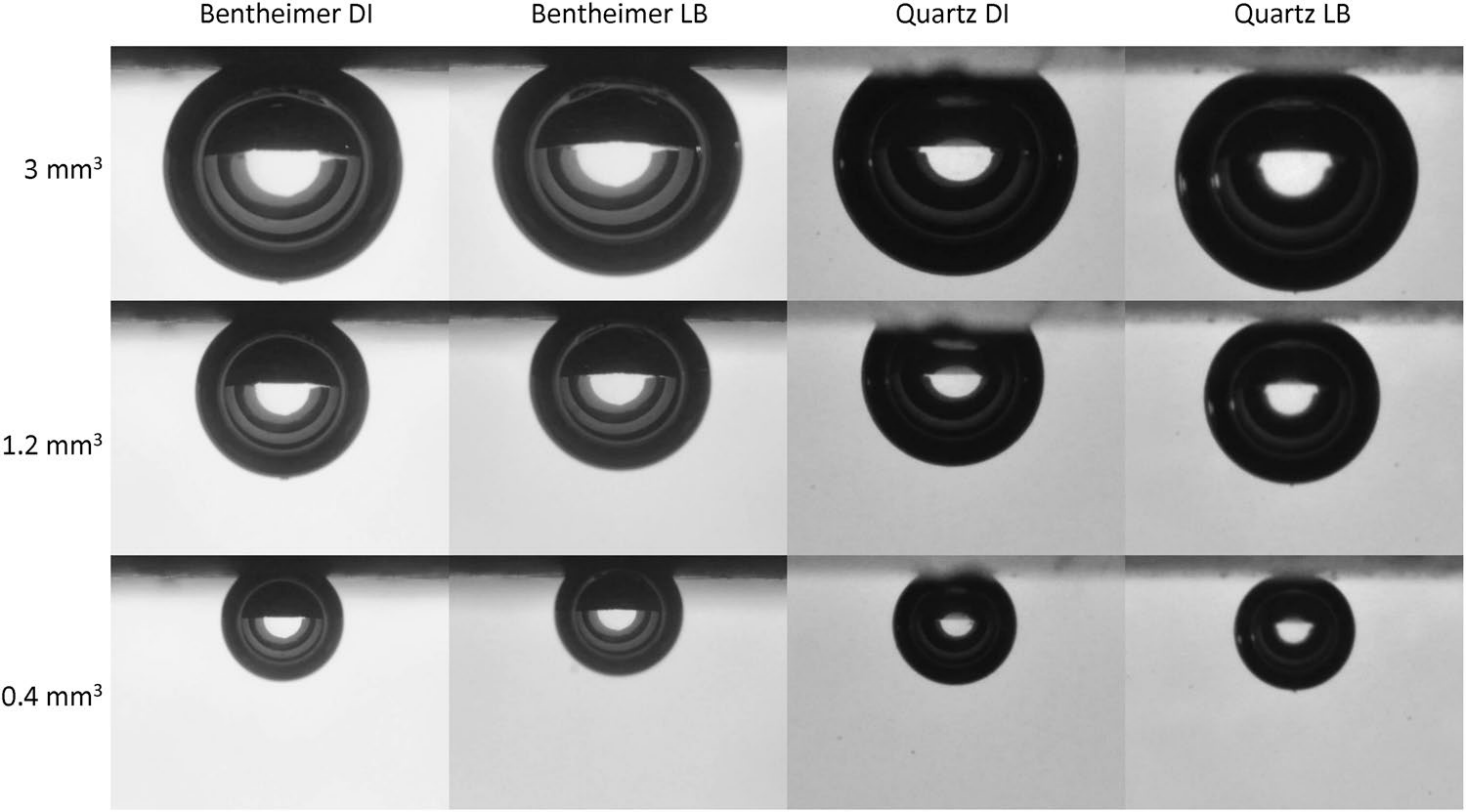
Hydrogen bubbles in touch with a Bentheimer Sandstone (left) and a pure Quartz sample (right) for both DI-water and at the end of the Living Brine (LB) experiment at volumes of 0.4, 1.2, and 3 mm^3^.

In both the experiment with the Bentheimer Sandstone and the experiment with the Quartz sample, high microbial activity has been observed, which led to the formation of a biofilm. In the case of the Bentheimer sandstone sample the biofilm did not impact the observed contact angles while the contact angles significantly decreased during the pure Quartz experiment making the system very water-wet. The Bentheimer Sandstone is almost completely comprised of Quartz^4,24^. Therefore, the main difference between the two systems is the surface roughness which is 0.03 mm for the “rough” Bentheimer Sandstone and 0.003 mm for the “smooth” Quartz sample. Also, note that the Bentheimer Sandstone sample is porous and the Quartz sample is non-porous. A possible explanation of the observed results is that in case of the Bentheimer Sandstone, pockets of brine exists in the valleys of the rough surface and the gas bubble is only in touch with the peaks i.e. a Cassie-Baxter type of surface coverage^28^. Although some of the brine in these valleys will be taken up by the “dry” hydrogen gas, the brine in these valleys will be replenished through the porous rock such that the surface conditions will stay the same while the hydrogen bubble is slowly dissolving into the brine. In the case of the non-porous smooth Quartz sample the system is different. Due to the smooth surface the pockets of brine will be much smaller, and eventually the brine will be taken up by the dry hydrogen gas and even the valleys will be filled with hydrogen gas i.e. a Wenzel surface coverage regime^28^. When this change in surface coverage regime from Cassie-Baxter to the Wenzel regime happens, the contact angle starts to decrease with decreasing volume (Fig. 4). In case of the Bentheimer sample the biofilm is formed within the valleys of the rough surface, and thereby not changing the solid surface that comes in touch with the hydrogen gas bubble, and no changes in apparent contact angle will be observed (Fig. 5). In the case of the smooth pure Quartz sample, the biofilm covers both peaks and valleys (Fig. 3). In this case, the gas bubble will come in touch with the biofilm (Fig. 5) and the apparent contact angle changes depending on the wettability of the biofilm, as well as the roughness of the texture of the biofilm. It is important to note that the translucency of the Quartz sample impacted the light reflection which caused a “cloudy shape” to appear around the bubble in the images, as can be seen in Fig. 5. This likely decreased the accuracy of the contact angle measurements for DI-water and brine. However, the formation of the biofilm made the Quartz sample more opaque and the cloudy shape disappeared. Therefore, for the living brine experiment an accuracy range of ± 3^∘^ can be expected. The roughness of the solid walls of pores in natural consolidated rock will predominantly be at the submicron scale^29^. Therefore, biofilms formed in natural consolidated porous rock will likely completely cover the pore surface and changes in the apparent wettability can be expected.

While the microfluidic study of Liu et al. 2023^11^, carried out at 35 bar and 37^∘^  C, observed a significant increase in apparent contact angle due to microbial activity, changing the conditions from water-wet to neutral-wet, the opposite was observed in this study where contact angles decreased due to the formation of the biofilm. In both studies sulphate reducing microbes were used, albeit a different group of bacteria. Biofilms consist of large amounts of microbes in a matrix of EPS. The overall wettability of the biofilm is mainly determined by the contents of EPS which can range from hydrophilic to hydrophobic. Hydrophilic biofilms could increase the storage injectivity and decrease the amount of hydrogen loss, while hydrophobic biofilms could have the opposite effect. The content of the EPS depends on the bacterial strain as well as the growth conditions in the system. Therefore, to determine the impact of microbial activity on the wettability during UHS will require accurate replication of the reservoir conditions including the chemical composition of the brine, the microbial community, as well as surface roughness, temperature, pressure and pH conditions.

## Conclusion

In this work, the impact of microbial activity on the wettability during Underground Hydrogen Storage was investigated by using the captive-bubble cell approach, a system where buoyancy and interfacial forces are the main acting forces. The experiments were carried out using two solid samples, a “rough” Bentheimer Sandstone and a “smooth” Quartz sample, living brine containing sulphate reducing microbes, and pure hydrogen gas. Our study showed that microbial activity can lead to the formation of a biofilm on the solid rock surface, however, whether this will lead to a change in wettability highly depends on the surface roughness of the solid surface. For the rough Bentheimer Sandstone the apparent contact angle was unchanged during the experiment, while for the smooth Quartz sample the apparent contact angle decreased significantly (making the system more water wet). This could be explained by the fact that, on rough surfaces, the biofilm forms within the crevices and the part of the solid surface in touch with the hydrogen bubble stays unchanged. On smooth surfaces the biofilm will cover the full surface and the wettability is altered as a result. Our observation of the decrease in apparent contact angle due to biofilm formation for the non-porous Quartz solid surface is in contrast with the reported results of a recent microfluidic study in the literature^11^. The wettability of the biofilm is mainly determined by the consistency of the EPS. The consistency of the EPS depend on the bacterial strain as well as the physical and chemical conditions of the system. Consequently, the impact of microbial activity on the wettability during UHS can only be determined when the reservoir conditions are accurately replicated.

### Supplementary Information

Supplementary Information.

## Data Availability

The datasets generated and analysed during the current study are available in the ADMIRE Public H2-Microbes-CapBubble repository, https://gitlab.tudelft.nl/ADMIRE_Public/H2-Microbes-CapBubble.

## References

[1] Hashemi L, Blunt M, Hajibeygi H (2021). Pore-scale modelling and sensitivity analyses of hydrogen-brine multiphase flow in geological porous media. Sci. Rep..

[2] Heinemann N (2021). Enabling large-scale hydrogen storage in porous media—The scientific challenges. Energy Environ. Sci..

[3] Laban M (2020). Hydrogen Storage in Salt Caverns: Chemical Modelling and Analysis of Large-Scale Hydrogen Storage in Underground Salt Caverns.

[4] Hashemi L, Glerum W, Farajzadeh R, Hajibeygi H (2021). Contact angle measurement for hydrogen/brine/sandstone system using captive-bubble method relevant for underground hydrogen storage. Adv. Water Resour..

[5] Zivar D, Kumar S, Foroozesh J (2021). Underground hydrogen storage: A comprehensive review. Int. J. Hydrogen Energy.

[6] Dopffel N, Jansen S, Gerritse J (2021). Microbial side effects of underground hydrogen storage—Knowledge gaps, risks and opportunities for successful implementation. Int. J. Hydrogen Energy.

[7] Thaysen EM (2021). Estimating microbial growth and hydrogen consumption in hydrogen storage in porous media. Renew. Sustain. Energy Rev..

[8] Payler SJ (2019). An ionic limit to life in the deep subsurface. Front. Microbiol..

[9] Al-Amshawee S (2021). Roughness and wettability of biofilm carriers: A systematic review. Environ. Technol. Innov..

[10] Gregory SP, Barnett MJ, Field LP, Milodowski AE (2019). Subsurface microbial hydrogen cycling: Natural occurrence and implications for industry. Microorganisms.

[11] Liu N, Kovscek AR, Fernø MA, Dopffel N (2023). Pore-scale study of microbial hydrogen consumption and wettability alteration during underground hydrogen storage. Front. Energy Res..

[12] Kowalewski E, Rueslåtten I, Steen KH, Bødtker G, Torsæter O (2006). Microbial improved oil recovery-bacterial induced wettability and interfacial tension effects on oil production. J. Petrol. Sci. Eng..

[13] Alkan H (2019). Investigation of spontaneous imbibition induced by wettability alteration as a recovery mechanism in microbial enhanced oil recovery. J. Petrol. Sci. Eng..

[14] Aslannezhad M (2023). A review of hydrogen/rock/brine interaction: Implications for hydrogen geo-storage. Prog. Energy Combust. Sci..

[15] Hashemi L, Boon M, Glerum W, Farajzadeh R, Hajibeygi H (2022). A comparative study for h2-ch4 mixture wettability in sandstone porous rocks relevant to underground hydrogen storage. Adv. Water Resour..

[16] Higgs S (2022). In-situ hydrogen wettability characterisation for underground hydrogen storage. Int. J. Hydrogen Energy.

[17] Iglauer S, Ali M, Keshavarz A (2021). Hydrogen wettability of sandstone reservoirs: Implications for hydrogen geo-storage. Geophys. Res. Lett..

[18] Ali M (2021). Influence of pressure, temperature and organic surface concentration on hydrogen wettability of caprock; implications for hydrogen geo-storage. Energy Rep..

[19] van Rooijen W, Hashemi L, Boon M, Farajzadeh R, Hajibeygi H (2022). Microfluidics-based analysis of dynamic contact angles relevant for underground hydrogen storage. Adv. Water Resour..

[20] Yekta AE, Manceau J-C, Gaboreau S, Pichavant M, Audigane P (2018). Determination of hydrogen-water relative permeability and capillary pressure in sandstone: Application to underground hydrogen injection in sedimentary formations. Transp. Porous Media.

[21] Boon M, Hajibeygi H (2022). Experimental characterization of h2/water multiphase flow in heterogeneous sandstone rock at the core scale relevant for underground hydrogen storage (uhs). Sci. Rep..

[22] Keller KL (2014). New model for electron flow for sulfate reduction in desulfovibrio alaskensis g20. Appl. Environ. Microbiol..

[23] Feio MJ (2004). Desulfovibrio alaskensis sp. nov., a sulphate-reducing bacterium from a soured oil reservoir. Int. J. Syst. Evol. Microbiol..

[24] Peksa AE, Wolf K-HAA, Zitha PLJ (2015). Bentheimer sandstone revisited for experimental purposes. Mar. Pet. Geol..

[25] Pan B, Yin X, Iglauer S (2021). Rock-fluid interfacial tension at subsurface conditions: Implications for h2, co2 and natural gas geo-storage. Int. J. Hydrogen Energy.

[26] Li D, Cheng P, Neumann AW (1992). Contact angle measurement by axisymmetric drop shape analysis (adsa). Adv. Coll. Interface Sci..

[27] Dopffel N (2023). Microbial hydrogen consumption leads to a significant ph increase under high saline conditions—Implications for hydrogen storage in salt caverns. Sc. iRep..

[28] Xiao Y, Zheng J, He Y, Wang L (2022). Droplet and bubble wetting behaviors: The roles of surface wettability and roughness. Colloids Surf. A.

[29] Lai P, Moulton K, Krevor S (2015). Pore-scale heterogeneity in the mineral distribution and reactive surface area of porous rocks. Chem. Geol..

