# Supplementary material for: Microbial induced wettability alteration with implications for Underground Hydrogen Storage
Source: Sci Rep. 2024 Apr 8;14:8248. doi: 10.1038/s41598-024-58951-6 (PMC11001864; doi:10.1038/s41598-024-58951-6)
Supplement: Supplementary file 1 — Supplementary Information. [file 41598_2024_58951_MOESM1_ESM.pdf]

Supplementary Information for "Microbial induced wettability alteration with implications for Underground Hydrogen Storage"

M.M. Boon[1,2*], I. Buntic[3], K. Ahmed[1], N. Dopffel[4], C. Peters[5] and H. Hajibeygi[1]

[1]Delft University of Technology, Faculty of Civil Engineering and Geosciences, Delft, 2600 GA, The Netherlands
[2]University of Stuttgart, Institute of Applied Mechanics, Stuttgart, 70569, Germany
[3]University of Stuttgart, Department of Hydromechanics and Modelling of Hydrosystems, Stuttgart, 70569, Germany
[4]NORCE Norwegian Research Centre AS, Bergen, 5008, Norway
[5]Department of Civil and Environmental Engineering, Princeton University, Princeton, New Jersey, USA.

*m.m.boon@tudelft.nl

Contact angles for solution with dead cells and solution with metabolites

Next to DI-water, brine, and living brine, contact angle measurements were made for the Bentheimer Sandstone system using a solution of dead cells in DI-water (DC) where the cells were dispersed in the solution, and a brine with metabolites but without microbes (MB) containing approximately 34 mM acetate. The experimental conditions of these experiments can be found in Table 1. The presence of metabolites or dead cells reduces the interfacial tension, decreasing the system's intrinsic wettability. For the living brine experiment with the smooth Quartz sample of this study, this led to a reduction in the apparent contact angle, however, for the living brine experiment with the Bentheimer sandstone sample, no changes in the apparent contact angles were observed due to the surface roughness.

Figure S1 shows the contact angle versus volume curves for hydrogen bubbles in touch with a Bentheimer sandstone rock for four different liquids: solution of dead cells in DI-water (DC), a brine with metabolites but without microbes (MB), living brine (LB), and DI-water (DI). It can be seen that similar results were obtained for each of the different liquids. The small shift in contact angles is due to the location of the bubbles, slightly higher contact angles are obtained for bubbles slightly right of the nozzle. This

shows that for rough samples such as the Bentheimer Sandstone in this study, changes in interfacial tension on the order of ~20 mN/m will not lead to observable changes in the contact angle.

Table S1: Experimental conditions for the Bentheimer Sandstone system using a solution of dead cells in DI-water (DC), and a brine with metabolites but without microbes (MB).

Liquid	pH	location wrt nozzle
**Bentheimer Sandstone**		
MB-1	7.9	center
MB-2	8.0	right
DC	6.1	right
LB (day 10)	7.8	right
DI	6.2	center

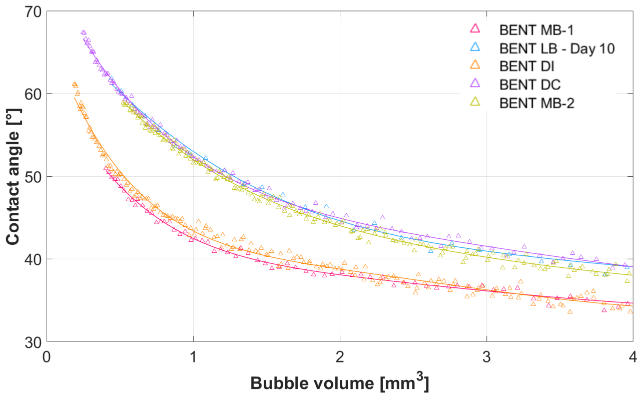

Figure S1: Contact angle versus volume for the Bentheimer Sandstone rock sample using a solution of dead cells in DI-water (DC), and a brine with metabolites but without microbes (MB). The curves for DI-water (DI) and living brine (LB) are shown for comparison.

Iron-sulfide formation due to microbial activity.

During the living brine experiment, Fe(II) which was present in the media and Bentheimer sandstone rock itself, reacted with the H_2S formed by the microbes. This resulted in the formation of iron-sulfide (FeS) which turned the Bentheimer rock sample dark (Figure 2).

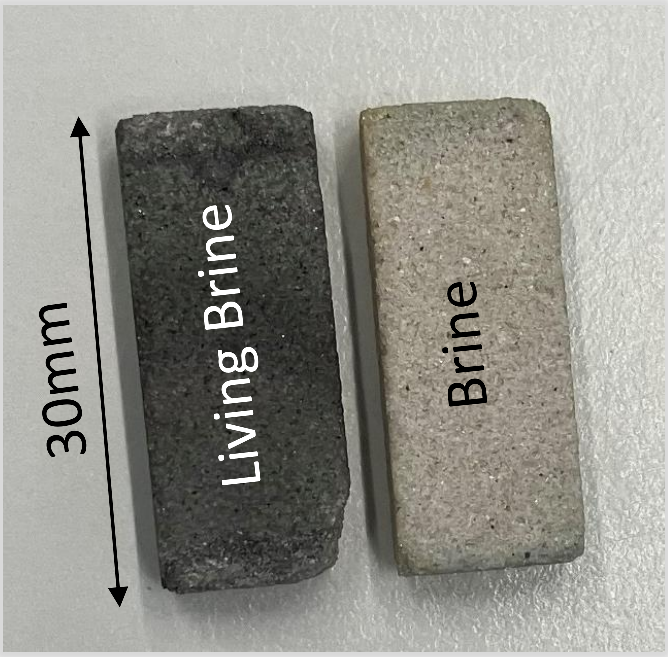

Figure S2: Bentheimer rock sample after a regular brine experiment (right) and after a living brine experiment (left). The sample turned black due to the formation of Iron Sulfide (FeS).
